# Behavioural effects of noise on Linnaeus’s two-toed sloth (*Choloepus didactylus*) in a walk-through enclosure

**DOI:** 10.1017/awf.2023.34

**Published:** 2023-05-19

**Authors:** Yuri Garcia de Abreu Rezende, Marina Bonde Queiroz, Robert John Young, Angélica da Silva Vasconcellos

**Affiliations:** 1 Department of Biological Sciences, Pontifical Catholic University of Minas Gerais, Brazil; 2 School of Science, Engineering and the Environment and Life Sciences, University of Salford, Salford, GB, United Kingdom of Great Britain and Northern Ireland

**Keywords:** Acoustic stress, animal welfare, behaviour, captivity, sound pressure, two-toed-sloth

## Abstract

Anthropogenic noise has been related to stress in captive animals; despite this there have been few studies on animal welfare assessment in walk-through zoo enclosures. We aimed to investigate the behavioural effects of noise on a male-female pair of two-toed sloths (*Choloepus didactylus*), housed in a walk-through enclosure in a zoo in the UK. The animals were filmed for 24 h per day, during three days per week, including days with potential low and high flow of visitors, for three weeks. Sound pressure measurement was performed four times each collection day (twice in the morning, once at noon and once in the afternoon), for 15 min per session, using a sound level meter. The number of visitors passing the enclosure during each session was also recorded. The videos were analysed using focal sampling, with continuous recording of behaviour. Correlations between noise and the behaviours expressed during, and in the 24 h after the acoustic recording, were investigated. The number of visitors correlated with the acoustic parameters. At the moment of exposure, higher levels of noise correlated with decreased inactivity, and longer expression of locomotion and maintenance behaviours for the male; the female spent more time inside a box in these moments. During the 24 h hours after exposure to loud noise, the female showed no behavioural changes while the male tended to reduce foraging. The behavioural changes observed in both individuals have already been reported in other species, in response to stressful events. Our study indicates the need for a good acoustic management in walk-through zoo enclosures where sloths are housed.

## Introduction

Animals housed in zoos are exposed to various stimuli that can impinge upon their welfare (Birke 2002; Cooke & Schillaci 2007; Clark *et al.* 2012; Maia *et al.* 2012). Among these stimuli there is exposure to visitors (Carder & Semple 2008; Clark *et al.* 2012; Farrand *et al.* 2014), which has been connected to the ‘zoo-visitor effect’ (e.g. Davey 2007). Such effect may be assessed through changes in behaviour and/or physiological responses of the animals, when exposed to zoo visitors (Davey 2006). However, research on visitor effect may lack scientific rigorousness as a result of constraints regarding the control of variables related to visitor presence (Farrand *et al.* 2014). Animals may perceive the presence of visitors via a variety of different perception channels: visual, olfactory and auditory (Young 2003). While visual and olfactory stimuli are difficult to measure, accurate quantification of auditory stimuli is feasible. Zoo visitor-noise pollution has been referred to as having a negative effect on animal welfare (Owen *et al.* 2004; Powell *et al.* 2006). Besides the effects of noise on the stress-response system (e.g. Bowles & Eckert 1997; Ward *et al.* 1999; Owen *et al.* 2004; Wysocki *et al.* 2006), noise has been reported to cause DNA damage, alterations in gene expression and numerous cellular processes with effects on neural, developmental, immunological and physiological functioning (Kight & Swaddle 2011).

Studies on visitor noise have reported detrimental effects on various species: pumas (*Puma concolor*: Maia 2009; Maia *et al.* 2012), gorillas (*Gorilla gorilla*: Clark *et al.* 2012), chimpanzees (*Pan troglodytes*) and spectacled bears (*Tremarctos ornatus*) reduced feeding, and increased locomotion (Noga 2010); Japanese monkeys (*Macaca fuscata*) increased reaction time during a cognitive task (Cronin *et al.* 2018), and bush dogs (*Speothos venaticus*) increased exhibition of stereotypies (Corat & Chierregatto 2015). Koalas (*Phascolarctos cinereus*) (Larsen *et al.* 2014) increased downtime and alertness; Western grey (*Macropus fuliginosus fuliginosus*) and red kangaroos (*Macropus rufus*) increased vigilance behaviours (Larsen *et al.* 2014; Sherwen *et al.* 2015a). Physiological effects, in tandem with such behavioural responses, have supported the interpretation of these responses as detrimental. For example, increased exhibition of stereotypies concomitant with increased glucocorticoid concentrations have been reported in several studies (e.g. Malmkvist *et al.* 2011; Shepherdson *et al.* 2013; Pizzutto *et al.* 2015; for a review, see Mason 1991). Giant pandas (*Ailuropoda melanoleuca*) showed increased locomotion, agitation, scratching, and glucocorticoid concentration when exposed to loud noise (Owen *et al.* 2004). Increased glucocorticoid concentration triggered by acoustic stressors may cause immunosuppression, insulin resistance, cardiovascular disease, catabolism (molecular decomposition), and intestinal problems (Spreng 2000).

Zoo-animal responses to visitors may depend upon species- and individual-characteristics, the nature of visitor-animal interactions, and enclosure design (Woolway & Goodenough 2017; Learmonth *et al.* 2018). Compared to traditional facilities (in which visitors remain outside), walk-through enclosures have greater potential to affect the welfare of animals, since contact with visitors (auditory, visual) is magnified, due to greater physical proximity and/or the absence of physical barriers between animals and visitors (Learmonth *et al.* 2018). However, such enclosures are increasingly popular, and their impacts on animal welfare are understudied (Sherwen *et al.* 2015b). The few studies addressing this type of housing report that visitors have an effect on animals: quokkas (*Setonix brachyurus*) spent more time hidden when the enclosure was open to visitors (Learmonth *et al.* 2018) and squirrels (*Sciurus vulgaris*: Woolway & Goodenough 2017) moved more and fed less. In contrast, Jones *et al.* (2016) pointed to a positive effect on crowned lemurs (*Eulemur coronatus*) via a decrease in aggression among conspecifics with an increase in numbers of visitors. The scarcity of data indicates the need for investigations into the effects of such enclosures on the welfare of animals.

The two-toed sloth (*Choloepus didactylus*) is a mid-sized, nocturnal tree mammal found in the rainforests of South America, which spends most of its time in the treetops (Eisenberg & Redford 1999; Nowak & Walker 1999; Bezerra 2008; Peery & Pauli 2012). Having very low metabolic rates (roughly half those of other placental mammals) and feeding on a low-energy diet, they require up to 14 h of daily inactivity, and locomotion occurs slowly (Montgomery & Sunquist 1975; Nagy & Montgomery 1980). In this study, we investigated the possible effects of visitor noise on the behaviour of two-toed sloths, housed in a walk-through zoo enclosure.

## Materials and methods

### Ethical permission

All procedures here were evaluated and approved by the Ethics Committee for Animal Use from the Pontifical Catholic University of Minas Gerais (Permit n 007/2014).

### Study protocol

One male and one female two-toed sloth, housed in a walk-through enclosure in a zoo in the UK were the subject of this study. Visitors were unable to touch the sloths in the enclosure,with animals remaining above them (at a height of approximately 6 m) for the majority of the time, i.e. hanging from ropes, without any barriers to isolate the animals from visitor noise. The enclosure was indoors, made of concrete and round in shape (approximately 11 m in diameter). The animals were fed in the mornings, prior to the admittance of visitors. Food was made available through environmental enrichment: feeding items were spread over the floor, and within the branches of trees in the enclosure. The animals were filmed for 24 h per day, for three consecutive days a week (Fridays, Saturdays and Sundays), during the first three weeks of July 2017. The videos were analysed through focal sampling with continuous recording of behaviour, using the Solomon Coder programme (Copyright 2006–2017 by András Péter). Behavioural observations were based on an ethogram (Table 1) adapted from Hayssen (2011), Silva *et al.* (2013), and Clark and Melfi (2012). Sound measurement was performed four times per day using the SVAN 977A sound level meter (SVAN 977A, SVANTEK, Poland). The equipment was fixed on a tripod, in the visitor space, facing the enclosure, 1.5 m above the floor and a good distance clear from the boundaries of the enclosure. Each session lasted 15 min, distributed between morning (0930–0945h; with the zoo still closed to visitors), mid-morning (1045–1100h), midday (1200–1215h) and afternoon (1500–1515h). During each session, the number of visitors passing through the enclosure was also assessed. Since there have been no studies to date assessing which aspects of sound have the greatest effect on animals, we chose to evaluate three parameters, using the A-weighting filter: Equivalent Continuous Noise Level (LA_eq_), Maximum Sound Pressure Level (LA_max_), and Peak Value (LA_peak_). The LA_eq_ is used to measure the average sound pressure levels and calculate the average noise level (energy) to which an environment is exposed (Duarte *et al.* 2011), LA_max_ is the maximum level of noise (mean square root) during a certain period and LA_peak_ is the highest point of raw sound pressure, without considering time. A-weighting was chosen because this filter has been tested previously, and was shown to correlate to sloths’ behaviour more than filter Z. This correlation suggests their acoustic sensitivity may be similar to ours (Queiróz 2018).

**Table 1.** Ethogram of *Choloepus didactylus* observed in this study*

Behavioural categories	Behaviours	Description
Inactivity	Resting	Still, with crouched body, does not seem to be vigilant
	Sitting	Still, with head up, does not seem to be vigilant
	Stationary	Body hung and motionless
Locomotion	Moving	Changing location, from one place to another
Maintenance	Moving without locomotion	Extension of a limb for no apparent reason, then returning to a rest position
	Scratching	Body rub using claws
	Licking	Self-grooming using the tongue
	Drinking	Ingestion of water
	Yawning	The upper and lower jaws are moved in opposite directions, opening the mouth
Foraging	Eating	Ingestion of food
	Food-handling	Food manipulation
	Inspecting	Visual investigation of the enclosure
Sexual behaviour	Copulating	The male is positioned dorsally in relation to the female
Affiliative interaction	Affiliative interaction	One arm is extended not aggressively towards conspecific
Agonistic interaction	Agonistic interaction	One arm is extended aggressively toward another sloth, followed by a scratch or bite
Non visible	Non visible	The animal is not visible to the observer, inside one of the sleeping boxes

* Adapted from Hayssen (2011), Silva *et al.* (2013) and Clark and Melfi (2012).

### Statistical analysis

For statistical analysis, we grouped the observed behaviours into the following categories: Inactivity; Locomotion; Maintenance; Foraging; Sexual behaviour; Affiliative interaction; Agonistic interaction; and Non-visible. Every behavioural category was correlated with each acoustic parameter (LA_eq_, LA_max_, LA_peak_) using Pearson’s correlation test for parametric data or Spearman’s correlation test for non-parametric data (Zar 2010). These analyses were performed for the behaviours exhibited during the 15-min of acoustic collection, and for the behaviours recorded during the subsequent 24 h, to assess a possible lasting effect of sound pressure. In the case of data correlation within 24 h of noise exposure, the behaviours recorded on video were correlated with the LA_eq_ recorded on the previous day. The number of visitors passing in the enclosure during the 15-min sessions were also correlated to the acoustic parameters. Considering all behaviour data were correlated with three acoustic parameters, we applied Bonferroni correction, and considered *P*-values which were not greater than 0.017 as significant.

## Results

**Figure 1.**
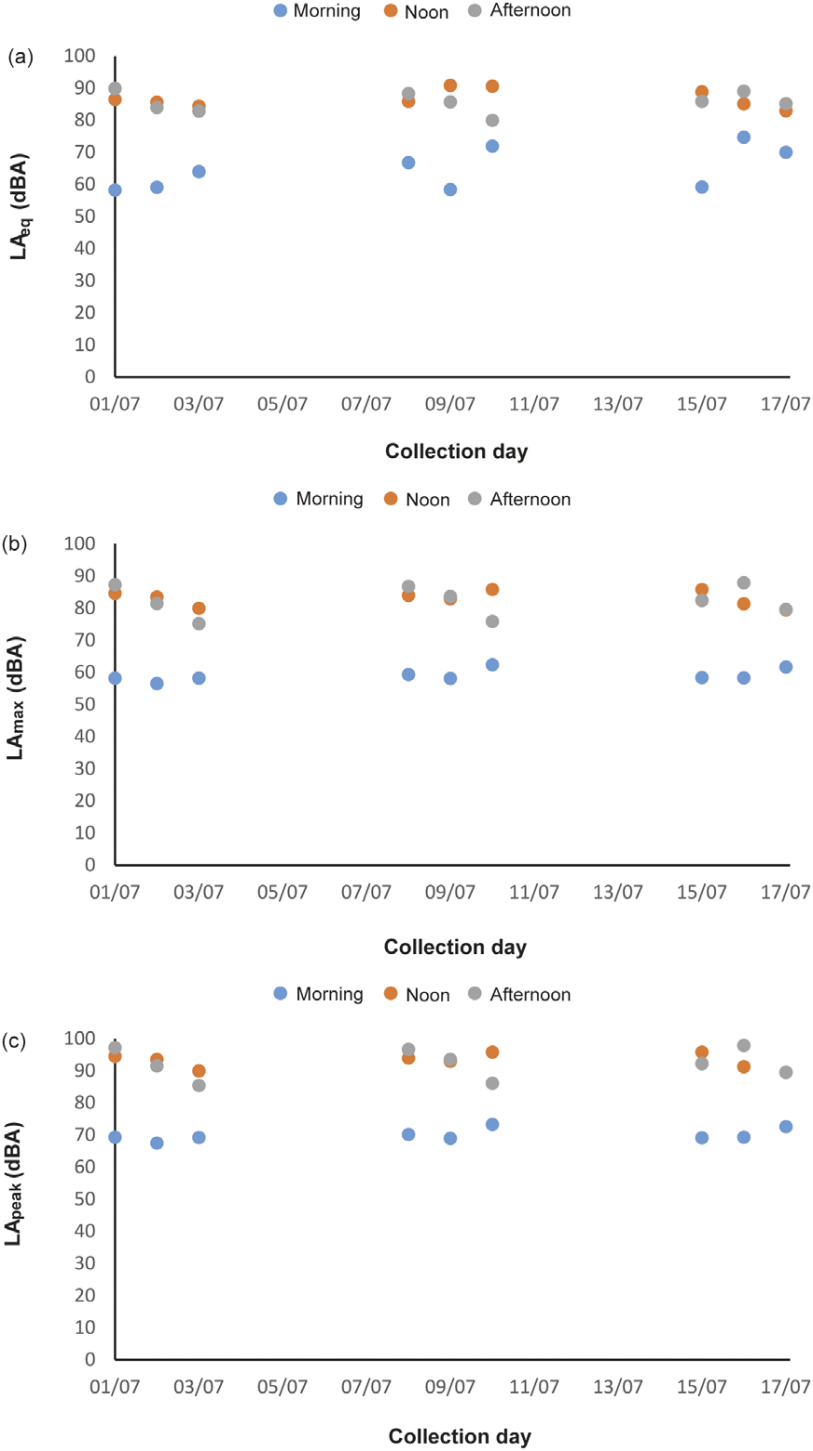
Mean acoustic parameters measured along three weeks, in three periods (morning, noon, and afternoon), in 15-minute sessions inside a walk-through enclosure of Choloepus didactylus, in a zoo in the United Kingdom. A – LAeq (equivalent continuous noise level); B – LAmax (maximum sound pressure level); C - LApeak (peak value).

**Figure 2.**
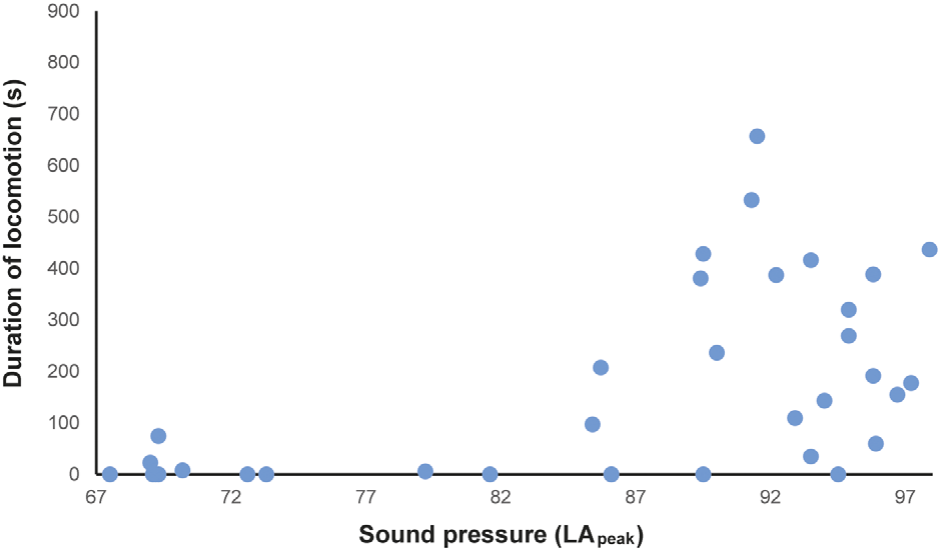
Duration (s) of locomotion of the male Choloepus didactylus housed in a walk-through enclosure, in a zoo in the UK, as a function of the noise level (LApeak) during the 15-min sessions of noise recording.

**Figure 3.**
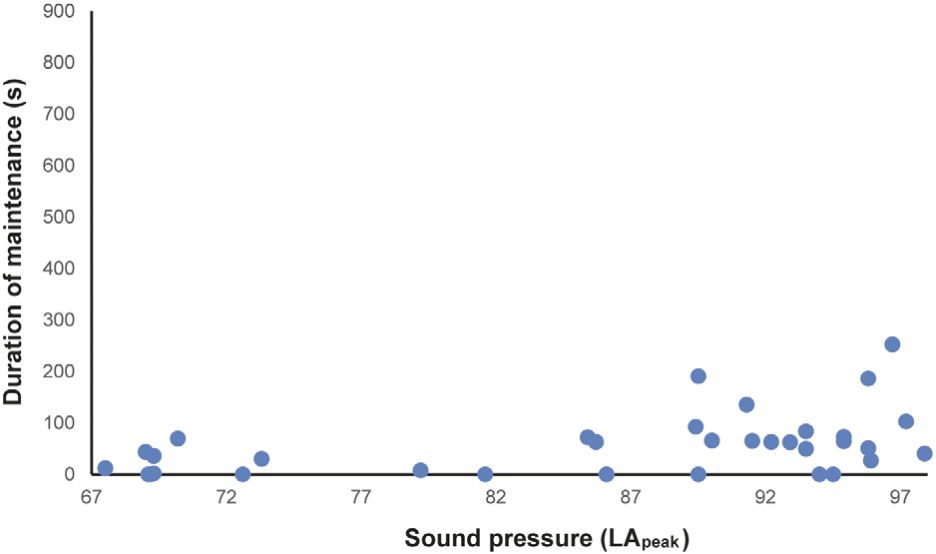
Duration (s) of maintenance behaviours of the male Choloepus didactylus housed in a walk-through enclosure, in a zoo in the UK, as a function of the noise level (LApeak) during the 15-min sessions of noise recording.

**Figure 4.**
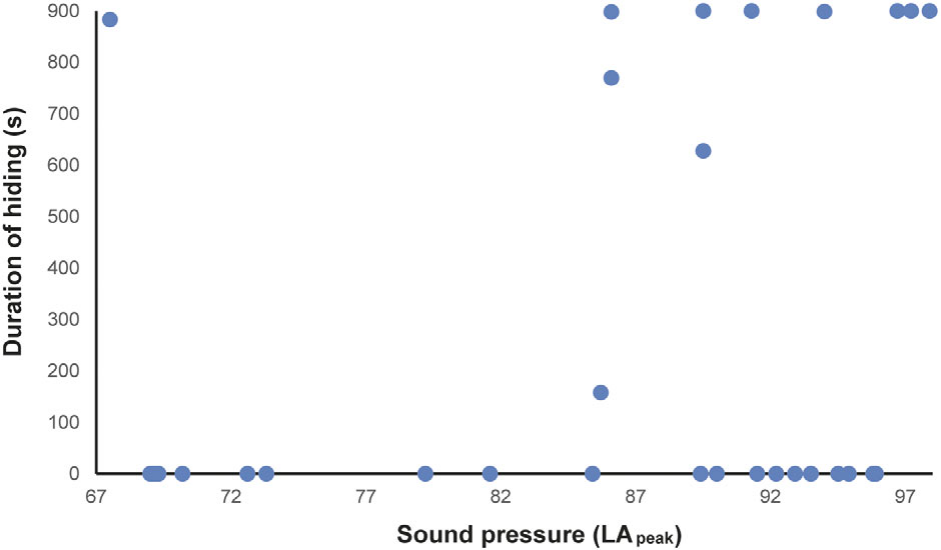
Duration (s) of the time the female Choloepus didactylus spent out of view, in a walk-through enclosure, in a zoo in the UK, as a function of the noise level (LApeak) during the 15-min sessions of noise recording.

**Figure 5.**
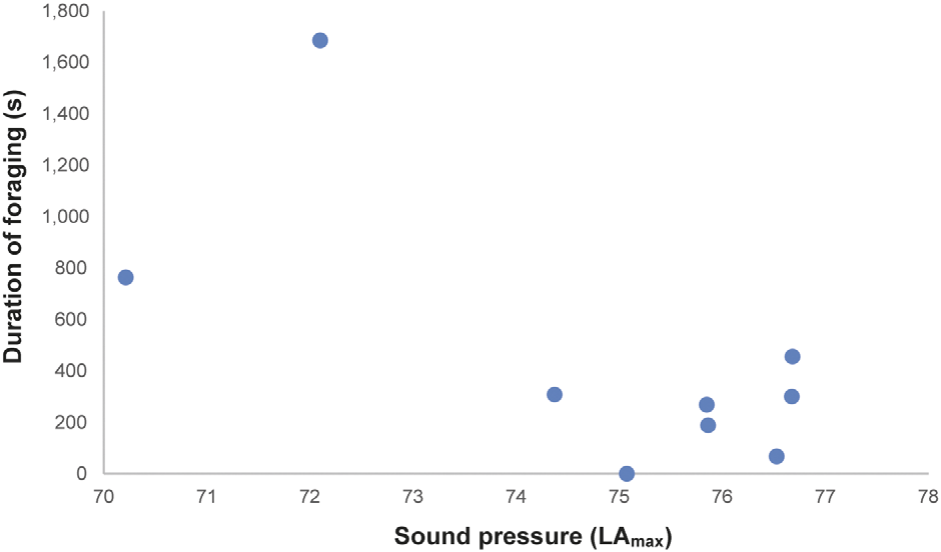
Duration (s) of foraging behaviour of the male Choloepus didactylus housed in a walk-through enclosure, in a zoo in the UK, as a function of the noise level (LAmax) within 24 h of noise exposure.

In total, 216 h of behavioural recording, and 36 × 15-min sessions of acoustic recording, were produced. The activity budget of male and female animals can be seen in the Supplementary material. The noise levels recorded ranged from 51.7 to 122.2 dBA (LA_peak_), with an LA_eq_ of 85.5 dBA. The acoustic parameters from the mornings (first acoustic collections, without visitors) were: LA_eq_ ranging from 58.2 to 74.7 dBA; LA_max_ ranging from 56.5 to 62.4 dBA; LA_peak_ from 67.5 to 73.3 dBA (Figure 1[a]–[c]). The number of visitors inside the enclosure during each 15-min session (ranging from 2 to 322 people) correlated strongly and positively with the three acoustic parameters (LA_peak_ *P* < 0.0001, Pearson = 0.92; LA_max_ *P* < 0.0001, Pearson = 0.92; LA_eq_ *P* < 0.0001, Spearman = 0.87). During exposure to increasing noise levels, we recorded significant reduction in inactivity (*P* = 0.0016, *r* = –0.5141 LA_peak_; *P* = 0.0015, *r* = –0.5158 LA_max_; *P* = 0.0011, *r* = –0.5273 LA_eq_), and increased locomotion (*P* = 0.0004, *r* = 0.5625 LA_peak_; *P* = 0.0004, *r* = 0.5626 LA_max_; *P* = 0.0019, *r* = 0.5071 LA_eq_; Figure 2) and maintenance behaviours (*P* = 0.0081, *r* = 0.4403 LA_peak_; *P* = 0.0083, *r* = 0.4389 LA_max_; *P* = 0.0149, *r* = 0.4082 LA_eq_; Figure 3) for the male. The female, when noise was higher, spent significantly more time inside a box (*P* = 0.0170, *r* = 0.4010 LA_peak_; *P* = 0.0170, *r* = 0.4006 LA_max_; *P* = 0.0149; Figure 4). Within 24 h of noise exposure, the female showed no behavioural changes but the male tended to spend less time foraging when LA_max_ was higher the previous day (*P* = 0.0479, *r* = –0.6709; Figure 5). For all other behaviours recorded, the correlations with acoustic parameters were not statistically significant (for more details, please see Tables S1 and S2, in the Supplementary material).

## Discussion

In this study, we evaluated the behaviours exhibited by a pair of two-toed sloths housed in a walk-through enclosure, on days with different levels of sound pressure – measured as LA_peak_, LA_max_ and LA_eq_ – due to human visitation. During exposure to higher sound pressure, the male moved more and spent longer time in maintenance behaviours while the female spent more time out of view. Within 24 h of experiencing intense noise exposure, the male displayed a tendency to reduce foraging.

An increase in locomotion, or in general activity in zoo animals – as observed in the male sloth in response to increased noise in this study – has been interpreted as indicative of improved welfare for certain species (e.g. lions [*Panthera leo*]: Novo & Santos 2014, capuchin monkeys [*Sapajus libidinosus*]: Koether 2017, jaguarundis [*Herpailurus yagouaroundi*]: Buhr *et al.* 2018, and Southern brown howlers [*Alouatta guariba clamitans*]: Muhle & Bicca-Marques 2008). However, studies with other species have shown a correlation between increased activity/locomotion and acoustic stress; in pumas (Maia 2009; Maia *et al.* 2012), gorillas (Clark *et al.* 2012), chimpanzees and spectacled bears (Noga 2010). In these cases, such increased activity was interpreted as an attempt by the animal to mitigate stress (Mitchell *et al.* 1992; Boere 2001; Hosey 2005), or simply as restlessness, caused by noise (Davey 2006; Quadros *et al.* 2014; Hashmi & Sullivan 2020). These apparently contradictory interpretations point to the need for a careful study of data, based on species characteristics (Queiroz & Young 2018) and, preferably, also on a joint evaluation based on technical measurements of sound pressure levels using appropriate equipment and protocols for a more accurate analysis (Quadros *et al.* 2014; Jakob-Hoff *et al.* 2019; Hashmi & Sullivan 2020).

The same debate pertaining to the interpretation of increased activity in zoo animals also applies regarding maintenance behaviours; the correlation of such behaviours with stress is often uncertain. Giant pandas increased locomotion, maintenance, scent-marking, and stereotypies during noisy periods but, according to the authors, there was no sign of a decline in welfare (Powell *et al.* 2006). However, white-handed gibbons (*Hylobates lar*) increased frequency and duration of scratching behaviour on days with more zoo visitors, especially children (Ribeiro 2016): in this case, scratching may be interpreted as a displacement behaviour (Roth & Cords 2020), suggestive of stress.

Here, we collected behavioural and acoustic data to carefully evaluate possible connections between noise and behaviour. Taking the species’ characteristics into account (low metabolic rates, low-energy diet, inactivity during most of the day, and slow locomotion; Montgomery & Sunquist 1975; Nagy & Montgomery 1980), the increased locomotion/activity and maintenance might be connected to acoustic stress. Another factor contributing to this interpretation is that the average noise level in the rainforest, natural habitat of the study species, is usually around 38 dB(A), considerably lower than the noise levels recorded in this study (Santos 2012). Besides, a study on visitor effect in zoos found that herbivorous species and those from closed habitats, such as ours here, were more negatively impacted by visitors than species from open habitats (Queiroz & Young 2018). The fact that the male has increased locomotion in the moments the noise was more intense during the day, and the records during the subsequent 24 h did not point to such an increase suggests the animal adjusted its activity budget, by relocating locomotion from their natural time (at night) to day-time. Such an adjustment may have unpredictable impacts on the welfare of this individual.

Although we have found no data on the hearing capacities of *C. didactylus*, studies on the ancestors of the sloth extant species point to a possible need for great sound sensitivity in the species (Blanco & Rinderknecht 2012; Blanco & Jones 2016). Those authors base their argument on the short snouts of certain sloth species, which suggest that they possess more focused and specialised sight, and as a consequence, a need for good sound acuity. This greater hearing sensitivity could make the species more prone to developing stress reactions when exposed to high levels of noise – such as those recorded in this study (e.g. up to 122.2 dBA). Apart from that, the potential (non-measured) high levels of reverberation can be also a factor affecting the sloths’ welfare. Both the circular shape of the enclosure and the material with which it was constructed favoured reverberation. Reverberation has been associated with impaired cognitive processes in humans (Kjellberg 2004), and interferences in animal communication (Padgham 2004). The disturbing effects of reverberation may also contribute to animal stress. Different responses to stress in males compared to females have previously been reported (e.g. Vasconcellos *et al.* 2009; Quadros *et al.* 2014). Such differing reactions might be due to diverse coping styles (e.g. Koolhaas *et al.* 1999; Ferreira *et al.* 2016).

In contrast to the apparently contradictory results mentioned previously, avoidance/hiding behaviours – as reported for the female in this study – have been consistently linked with fear, stress or apathy (Young 2003; Forkman *et al.* 2007; Sherwen *et al*. 2015c). Little penguins (*Eudyptula minor*) increased their distance from the visitor area and spent more time hiding in the presence of visitors (Sherwen *et al.* 2015c). Our data also corroborates studies with jaguarundis (Buhr 2018) and quokkas (Learmonth *et al.* 2018) in which hiding behaviours were correlated with visitor presence, and the noise they created.

Foraging behaviours are an important component of the behavioural repertoire of any species, since they are essential for survivorship (Young 2003). A reduction in the exhibition of foraging in response to visitor noise has been also reported in pumas (Ricci *et al.* 2018) and tigers (*Panthera tigris*: Kerley *et al.* 2002). On visiting days, a giant otter (*Pteronura brasiliensis*) showed lower frequency of eating, aquatic activity, playing and rolling, behaviours that tend to be associated with good welfare (Oliveira & Carpi 2016).

Although some behavioural alterations reported in this study could lead to conflicting interpretations if taken in isolation (increase in locomotion and maintenance) when seen as a whole, and considering species’ characteristics, our data suggest that our study animals were in a state of restlessness due to noise – a condition with the potential to impact negatively on welfare. Such results have already been reported in walk-through enclosures for squirrels (Woolway & Goodenough 2017). The possible restlessness, in conjunction with an increase in hidden (out of view) time for the female and the tendency for decreased foraging as a medium-term noise response for the male, suggests a stress response, which can be related to reduced welfare. Discomfort due to interactions with visitors has already been reported for sloths (brown three-toed sloths [*Bradypus variegatus*]), with individuals performing vigilance and limb stretching, behaviours either not reported for the species, or performed at lower rates in the wild (Carder *et al.* 2018). Such results have also been interpreted by those authors as possible evidence of fear and stress.

## Animal welfare implications and conclusion

Although our results cannot be generalised due to our small sample size, they suggest the maintenance of two-toed sloths in walk-through enclosures – without any acoustic control (i.e. sonic barrier or management of visitor behaviour) – might be detrimental to their welfare. Acoustic management of zoo enclosures could include shorter visitation times and/or control of the number of concomitant visitors.

## Supplementary material

For supplementary material accompanying this paper visit https://doi.org/10.1017/awf.2023.34. click here to view supplementary material
