# Supplementary material for: Behavioural effects of noise on Linnaeus’s two-toed sloth (Choloepus didactylus) in a walk-through enclosure
Source: Anim Welf. 2023 May 19;32:e40. doi: 10.1017/awf.2023.34 (PMC10936293; doi:10.1017/awf.2023.34)
Supplement: Supplementary file 1 [file S0962728623000349sup001.docx]

**Supplementary Material**

| **Table S1 - Statistical results of the correlations (Pearson or Spearmann) between the acoustic parameters (LApeak, LAmax and LAeq) and the behavioural categories recorded during the 15-minute acoustic measurements in the study of *Choloepus didactylus* housed in a walk-through enclosure, in a zoo in the United Kingdom.** | | | | | | |
| --- | --- | --- | --- | --- | --- | --- |
| **Behavioural Category** | **LApeak** | | **LAmax** | | **LAeq** | |
|  | **P** | **r** | **P** | **r** | **P** | **r** |
| Inactivity | 0.931 F  0.002 M | -0.02 (P)  -0.51 (P) | 0.924 F  0.001 M | -0.02 (P)  -0.52 (P) | 0.445 F  0.001 M | -0.13 (P)  -0.53 (P) |
| Locomotion | 0.867 F  <0.001 M | 0.03 (S)  0.56 (P) | 0.884 F  <0.001 M | 0.03 (S)  0.56 (P) | 0.676 F  0.002 M | 0.07 (S)  0.51 (P) |
| Maintenance | 0.479 F  0.008 M | 0.12 (S)  0.44 (P) | 0.478 F  0.008 M | 0.12 (S)  0.44 (P) | 0.379 F  0.015 M | 0.15 (S)  0.41 (P) |
| Foraging | 0.811 F  0.216 M | -0.04 (S)  -0.21 (S) | 0.831 F  0.193 M | -0.04 (S)  -0.23 (S) | 0.745 F  0.183 M | -0.06 (S)  -0.23 (S) |
| Sexual behaviour | 0.436 F  0.436 M | -0.14 (S)  -0.14 (S) | 0.436 F  0.436 M | -0.14 (S)  -0.14 (S) | 0.436 F  0.436 M | -0.14(S)  -0.14 (S) |
| Affiliative interaction | 0.445 F  0.403 M | 0.13 (S)  0.15 (S) | 0.472 F  0.432 M | 0.13 (S)  0.14 (S) | 0.237 F  0.201 M | 0.20 (S)  0.22 (S) |
| Agonistic interaction | 0.560 F  0.560 M | -0.10 (S)  -0.10 (S) | 0.560 F  0.560 M | -0.10 (S)  -0.10 (S) | 0.771 F  0.771 M | -0.05 (S)  -0.05 (S) |
| Non-visible | 0.017 F  0.254 M | 0.40 (S)  0.20 (S) | 0.017 F  0.254 M | 0.40 (S)  0.20 (S) | 0.069 F  0.222 M | 0.31(S)  0.21 (S) |

*M – Male / F – Female

**S – Spearman / P – Pearson

| **Table S2 - Statistical results of the correlations (Pearson or Spearmann) between the acoustic parameters (LApeak, LAmax and LAeq) and the behavioural categories recorded during the 24 hours after acoustic measurements in the study of *Choloepus didactylus* housed in a walk-through enclosure, in a zoo in the United Kingdom.** | | | | | | |
| --- | --- | --- | --- | --- | --- | --- |
| **Behavioural Category** | **LApeak** | | **LAmax** | | **LAeq** | |
|  | **P** | **r** | **P** | **r** | **P** | **r** |
| Inactivity | 0.217 F  0.075 M | -0.46 (P)  -0.62 (P) | 0.197 F  0.063 M | -0.47 (P)  -0.64 (P) | 0.371 F  0.252 M | 0.34 (P)  0.43 (P) |
| Locomotion | 0.952 F  0.085 M | 0.02 (P)  0.60 (P) | 0.971 F  0.086 M | 0.01 (P)  0.60 (P) | 0.463 F  0.990 M | 0.28 (P)  -0.005 (P) |
| Maintenance | 0.822 F  0.285 M | 0.09 (P)  0.40 (P) | 0.843 F  0.288 M | 0.08 (P)  0.40 (P) | 0.548 F  0.981 M | 0.23 (P)  0.009 (P) |
| Foraging | 0.443 F  0.058 M | 0.29 (P)  -0.65 (P) | 0.437 F  0.050 M | 0.30 (P)  -0.67 (P) | 0.505 F  0.577 M | -0.26 (P)  0.22 (P) |
| Sexual behaviour | 0.270 F  0.270 M | -0.41 (S)  -0.41 (S) | 0.270 F  0.270 M | -0.41(S)  -0.41 (S) | 0.948 F  0.948 M | 0.02(S)  0.02 (S) |
| Affiliative interaction | 0.318 F  0.322 M | 0.38 (P)  0.37 (P) | 0.316 F  0.320 M | 0.38 (P)  0.37 (P) | 0.870 F  0.875 M | 0.60 (P)  0.06 (P) |
| Agonistic interaction | 0.194 F  0.194 M | 0.48 (S)  0.48 (S) | 0.194 F  0.194 M | 0.48 (S)  0.48 (S) | 0.270 F  0.270 M | 0.41 (S)  0.41 (S) |
| Non-visible | 0.374 F  0.735 M | -0.34 (P)  0.13 (P) | 0.363 F  0.713 M | -0.35 (P)  0.14 (P) | 0.488 F  0.603 M | -0.27(P)  -0.20 (P) |

*M – Male / F – Female

**S – Spearman / P – Pearson

Figure S1

a)

b)

**Figure S1 – Activity budget of the male (a) and the female (b) *Choloepus didactylus* housed in a walk-through enclosure, in a zoo in the United Kingdom. Data collected during nine days, with different intensities of visitation.**
